# Differential Protein Expression in Striatal D1- and D2-Dopamine Receptor-Expressing Medium Spiny Neurons

**DOI:** 10.3390/proteomes8040027

**Published:** 2020-10-13

**Authors:** M. Shahid Mansuri, Gang Peng, Rashaun S. Wilson, TuKiet T. Lam, Hongyu Zhao, Kenneth R. Williams, Angus C. Nairn

**Affiliations:** 1 Yale/NIDA Neuroproteomics Center, New Haven, CT 06511, USA; mohammad.mansuri@yale.edu (M.S.M.); rashaun.wilson@yale.edu (R.S.W.); tukiet.lam@yale.edu (T.T.L.); 2 Molecular Biophysics and Biochemistry, Yale University School of Medicine, New Haven, CT 06511, USA; 3 Department of Biostatistics, School of Public Health, Yale University, New Haven, CT 06520, USA; gang.peng@yale.edu (G.P.); hongyu.zhao@yale.edu (H.Z.); 4 Department of Genetics, School of Medicine, Yale University, New Haven, CT 06520, USA; 5 W.M Keck Biotechnology Resource Laboratory, Yale University School of Medicine, New Haven, CT 06511, USA; 6 Connecticut Mental Health Center, Department of Psychiatry, Yale School of Medicine, New Haven, CT 06511, USA

**Keywords:** dopamine receptor, D1 and D2 receptor, striatum, medium spiny neuron, cell-type-specific proteomics, FACs, FANs, TMT, PCT, quantitative mass spectrometry

## Abstract

Many neurological disorders and diseases including drug addiction are associated with specific neuronal cell types in the brain. The striatum, a region that plays a critically important role in the development of addictive drug-related behavior, provides a good example of the cellular heterogeneity challenges associated with analyses of specific neuronal cell types. Such studies are needed to identify the adaptive changes in neuroproteomic signaling that occur in response to diseases such as addiction. The striatum contains two major cell types, D1 and D2 type dopaminoceptive medium spiny neurons (MSNs), whose cell bodies and processes are intermingled throughout this region. Since little is known about the proteomes of these two neuronal cell populations, we have begun to address this challenge by using fluorescence-activated nuclear sorting (FANS) to isolate nuclei-containing fractions from striatum from D1 and D2 “Translating Ribosome Affinity Purification” (TRAP) mice. This approach enabled us to devise and implement a robust and reproducible workflow for preparing samples from specific MSN cell types for mass spectrometry analyses. These analyses quantified at least 685 proteins in each of four biological replicates of 50 K sorted nuclei from two D1 mice/replicate and from each of four biological replicates of 50 K sorted nuclei from two D2 mice/replicate. Proteome analyses identified 87 proteins that were differentially expressed in D1 versus D2 MSN nuclei and principal component analysis (PCA) of these proteins separated the 8 biological replicates into specific cell types. Central network analysis of the 87 differentially expressed proteins identified Hnrnpd and Hnmpa2b1 in D1 and Cct2 and Cct7 in D2 as potential central interactors. This workflow can now be used to improve our understanding of many neurological diseases including characterizing the short and long-term impact of drugs of abuse on the proteomes of these two dopaminoceptive neuronal populations.

## 1. Introduction

The striatum plays an important role in a wide range of neurological disorders including obsessive-compulsive disorder, Huntington’s and Parkinson’s disease, and drug addiction [1,2,3,4,5,6]. Substance abuse is one of several neurological diseases that involves specific cell types in the brain. For example, psychostimulants such as cocaine block the reuptake of dopamine leading to increased synaptic concentrations of neurotransmitter. Subsequently, this leads to aberrant signaling in specific sub-types of the γ-aminobutyric acid producing (GABAergic) medium-sized spiny neurons (MSNs) that comprise 95% of the neurons of the striatum [5,7]. Although morphologically similar, there are two large sub-types of striatal MSNs that differ in their projection patterns as well as in their expression of D1- or D2-classes of dopamine receptors [8]. Although the “direct” pathway dMSNs express relatively high levels of the D1 dopamine receptor (D1R), “indirect” pathway iMSNs express high levels of the D2 dopamine receptor (D2R) [8]. D1 and D2 dopamine receptors are differentially coupled to either increased or decreased cAMP signaling, respectively. Thus, exposure to psychostimulants results in opposite patterns of phosphorylation by cAMP-dependent protein kinase of important intracellular targets such as DARPP-32 in intermixed sub-populations of MSNs [9]. Biochemical analysis of striatum, in the absence of separation of different MSN cell types, leads to an averaging of the increased or decreased signals, and a loss of important information [10,11]. 

Since D1R- and D2R-MSNs, as with many other of the 500–1000 types of neurons and glia, exhibit distinct patterns of gene expression [12,13], it is essential to develop complementary approaches to enable quantitative MS/proteomic analyses of specific neuronal cell types and their organelles and sub-cellular compartments. These approaches will further our understanding of changes in protein expression or localization, as well as of differential post-translational protein modifications by obviating dilution effects from analyzing multiple cell types together and by improving signal to noise ratios for proteins in the targeted cell type [10,11]. Isolation of specific neural cell types is challenging due to their nonuniformity, intermingling, and complex projections to different brain regions. Two approaches that are being used to address this challenge are laser capture microdissection (LCM) [14], and fluorescence-activated cell (FACS) [15,16,17] and nuclear (FANS) [18,19,20] sorting in conjunction with transgenic and viral tools to isolate cell type- and organelle-specific proteomes. A central feature of many of these methods is the use of transgenic mouse lines that express fluorescent proteins as affinity tags on specific proteins in specific neuronal cell types. These include Translating Ribosome Affinity Purification (TRAP) lines that were designed to profile mRNA translation in defined cell types [21,22] and that allow isolation of nuclei from specific cell types using FANS [18]. TRAP analyses revealed that dMSNs expressing the D1R have vastly different translational profiles than the iMSNs that express the D2R [21]. Moreover, FANS analyses demonstrated that cocaine induces a significantly different pattern of histone modification in these two populations of mouse MSNs [18]. 

Although TRAP-based translational profiling and other single-cell- or single-nuclei-RNA sequencing methods provide a massively parallel approach to mRNA expression analyses of individual types of neural cells, these approaches do not account for differential control of protein synthesis, degradation, and post-translational modification. As a result, there often is relatively poor correspondence between transcriptomic and proteomic expression [23]. The poor correspondence between mRNA and protein expression in neural tissues was reinforced by an in-depth proteomic survey of the human brain that compared fold-changes in protein and mRNA levels across brain regions [24]. In general, this study found more pronounced inter-regional differential expression at the protein level which highlights the critical importance of MS/proteomics analyses. Surprisingly, and as noted previously [10], the proteomes of the two major types of striatal MSNs have yet to be effectively compared. To address this challenge, we have begun to compare the proteomes of D1R versus D2R-containing MSNs from mouse. 

Since the cell bodies and processes of these two major neuronal cell types are intermixed throughout the striatum, which complicates their clean isolation and downstream proteomic profiling, we used FANS to circumvent this problem. Although FACS is the most commonly used technology to isolate discrete populations of individual cell types [15,16,17], this approach may not be an optimal strategy to isolate intact neurons as the cells first need to be individually dissociated into a single-cell suspension [10]. During this process it is likely that synapses and neurites may become damaged or retract [10], a conclusion supported by our preliminary studies. Indeed, the papain digestion that is often used to break neural cell-cell interactions destroys many axons and dendrites that can stress neural cells and change their expression profiles [16]. To overcome this challenge, we used FANS to isolate nuclei-containing cellular fractions from striatum of D1R- and D2R-TRAP mice. This approach enabled us to carry out a robust and reproducible sample preparation workflow for downstream mass spectrometry studies. Since the amounts of protein that can be isolated with this approach are limiting, we coupled FANS isolation with a streamlined protocol for protein profiling that incorporates on-column Tandem Mass Tag (TMT) labeling [25]. Using this method, we quantified 714 proteins. Of the 685 proteins that did not have any missing values in any of the four D1 and four D2 biological replicates, 87 were significantly differentially regulated between these two cell types with 40 up-regulated and 47 down-regulated in D1 MSNs. Hierarchical clustering of the 87 differentially expressed proteins separated all eight samples into D1 and D2 clusters. GO term analysis of these proteins determined that 53% were derived from the nucleus. Central network analysis of the differentially expressed proteins identified different central interactors for each cell type: Hnrnpd and Hnrnpa2b1 in D1 and Cct2 and Cct7 in the D2 nuclear fractions. 

## 2. Materials and Methods

### 2.1. Animals

In all experiments, mice were between 4–8 weeks old and were maintained on a 12 h light/dark cycle. Transgenic Drd1a:EGFP-L10a or Drd2:EGFP-L10a bacTRAP lines were maintained on a C57BL/6J background. The eight D1 and eight D2 mice were killed by rapid decapitation and striatal tissue was rapidly dissected. After pooling the tissue obtained from two mice as described below, the resulting four D1 and four D2 biological replicates were frozen on dry ice before being homogenized and subjected to FANS isolation. Animals for all studies were handled in accordance with Yale IACUC and NIH guidelines (approval code: 10922 May 2020). They are in accordance with FELASA guidelines and the National law for Laboratory Animal Experimentation

### 2.2. Nuclei Isolation and FANS

Nuclear fractionation was carried out as described [18] with some modifications. Briefly, individual striata from each mouse were minced 100 times vertically and horizontally on a glass plate and briefly fixed with 4% (wt./vol) paraformaldehyde for 5 min on ice. The cross-linked tissues from two mice were pooled together and then homogenized in 500 μL of cold homogenization buffer (250 mM sucrose, 25 mM KCl, 5 mM MgCl_2_, 10 mM tricine pH 8, 100 mM NH_4_Cl, 1 μM DTT, protease inhibitor mixture (cOmplete™, Mini Protease Inhibitor Cocktail, MilliporeSigma, Burlington, MA, USA), phosphatase inhibitor mixture (PhosSTOP; MilliporeSigma)) by using 10 strokes with a loose pestle followed by 10 strokes with a tight pestle in a 2 mL Dounce homogenizer. The homogenate was centrifuged in a tabletop centrifuge at 2000× *g* for 5 min at 4 °C and the supernatant was discarded. After the pellet was resuspended with 1 mL of density medium (25% iodixanol (OptiPrep; Axis-Shield), 25 mM KCl, 5 mM MgCl_2_, 10 mM tricine-KOH pH 8), it was then overlaid on top of 200 μL of a 29% (*v*/*v*) iodixanol cushion. The nuclei were collected by centrifugation at 13,500× *g* for 30 min at 4 °C in a swinging bucket rotor (Beckman TL-100 ultracentrifuge; TLS55 rotor, Beckman Coulter, Indianapolis, IN, USA). After ultracentrifugation, the top lipid layer was removed with a pipette tip and then approximately 800 μL of the gradient medium was also removed and discarded. The nuclear pellet was washed three times with 1 mL of resuspension buffer (0.25 M sucrose, 25 mM KCl, 5 mM MgCl_2_, 20 mM tricine-KOH pH 7.8) in a siliconized Eppendorf tube by using tabletop centrifugation at 2000× *g* for 5 min at 4 °C. 

The four D1 and four D2 nuclei pellets were each resuspended in 250 μL resuspension buffer with DyeCycle Ruby (1:1000; Invitrogen) for 1 hr on ice. Nuclei were then sorted with a BD FACSAria™ cell sorter equipped with 640-nm and 488-nm excitation lasers and an 85 μm nozzle. Nuclei were gated by two criteria: the presence of a GFP signal above the background fluorescence level (as assessed by comparison with nuclei obtained from a wild-type littermate mouse) and the signal from DyeCycle Ruby corresponding to single nuclei. Sorted nuclei (i.e., about 80,000 for each biological replicate from two mice) were collected in 100 μL of resuspension buffer, centrifuged for 5 min at 2000× *g*, snap frozen in liquid nitrogen, and stored at −80 °C. To assess the purity of the isolated nuclei, the sorted nuclei were imaged at 40× with a fluorescence microscope using 640-nm and 488-nm excitation lasers. 

### 2.3. Immunoblotting of Nuclei

Sorted nuclei from D1-GFP^+^ or D2-GFP^+^ striatal tissues were lysed in 4% SDS and then heated for 15 min at 95 °C. The lysates containing 2–5 μg protein each were separated on 4–20% SDS-PAGE gradient gels (Bio-Rad, Hercules, CA, USA) and proteins were then transferred to PVDF. The PVDF membranes were blocked with 5% dried milk in 0.05% *v*/*v* phosphate buffered saline with Tween 20 (PBST) for 1 h prior to overnight incubation with primary antibodies (GFP, RRID AB_305564, 1:1000 or DARPP-32, RRID AB_2811192, 1:5000) at 4 °C with shaking. Blots were washed three times with 0.05% *v*/*v* PBST and then incubated with fluorescent secondary antibody at 1:10,000 dilution in PBST (0.5% (*v*/*v*)) for 30 min at room temperature. Blots were imaged using a LI-COR Odyssey Imaging System (LI-COR Biosciences, Lincoln, NE, USA). 

### 2.4. Sample Preparation for LC-MS/MS/MS

#### 2.4.1. Pressure-Cycling Technology (PCT)-Based Lysis and Digestion

Nuclei lysis and protein digestion were performed with a PCT-MicroPestle/Caps method in a Barocycler 2320 EXT (Pressure BioSciences, Inc., South Easton, MA, USA) as described [26]. The sorted nuclei (i.e., 50,000 of the approximately 80,000 that were obtained from the two mice that were used for each biological replicate) were first placed in microTubes (Pressure BioSciences, Easton, MA, USA) with a PCT-MicroPestle (Pressure BioSciences) in 30 μL of lysis buffer (100 mM triethylammonium bicarbonate (TEAB), 0.2% RapiGest, 10 mM tris(2-carboxyethyl) phosphine hydrochloride (TCEP) supplemented with cOmplete™ protease inhibitor cocktail (Roche, Basel, Switzerland) and PhosSTOP phosphatase inhibitor cocktail (Roche, Basel, Switzerland). Although cOmplete™ protease inhibitor was also included in the PCT/trypsin digest carried out by Shao et al. [26], investigators may consider omission of this protease inhibitor cocktail from the nuclei lysis and digestion buffer as it may decrease the active trypsin concentration. PCT-MicroPestle samples were processed with 60 cycles of 50 sec at high pressure (45,000 psi) followed by 10 sec at atmospheric pressure at 95 °C for 1 hr in the barocycler followed by reduction and alkylation for 30 min in the dark at room temperature. Thereafter, the PCT-MicroPestle was replaced with a PCT-MicroCap (50 μL size) and samples were digested with trypsin (Promega, Madison, WI, USA) at a concentration of 20 ng/μL. Digestion was performed in the barocycler at 37 °C using 360 cycles, each consisting of 50 s at 25,000 psi (high pressure) and 10 sec at atmospheric pressure for 6 h. After digestion was stopped by adding trifluoroacetic acid to a final concentration of 1%, the samples were ready for on-column TMT labeling.

#### 2.4.2. On-Column TMT Labeling

Stage tips were packed with two punches of C18 mesh (Empore) by using the blunt end of a 16-gauge needle as described [25], and were then conditioned with 50 μL methanol (MeOH), followed by 50 μL 50% acetonitrile (ACN)/0.1% TFA, and finally, the tips were equilibrated three times with 75 μL 0.1% TFA. The nuclei digest was loaded onto the C18 resin by spinning at 3500× *g*. Thereafter, 100 μL freshly prepared HEPES, pH 8, with 2 μL of TMT reagent (from stock) was passed over the C18 resin at 350× *g* until the entire solution had passed through. After TMT labeling, the C18 resin was washed twice with 75 μL 0.1% TFA and peptides were eluted with 50 μL 50% ACN/0.1% TFA followed by a second elution with 50% ACN/20 mM ammonium formate (NH_4_HCO_2_), pH 10. After using 5% of the elution volume to determine labeling efficiency, the samples were mixed prior to fractionation and analysis.

#### 2.4.3. Stage Tip bSDB Fractionation

200 μL pipette tips were packed with two punches of sulfonated divinylbenzene (SDB-RPS, Empore) with a 16-gauge needle and conditioned and equilibrated as described above. After loading 3 μg peptides, the resin was washed with 50 μL 20 mM NH_4_HCO_2_, pH 10. Then, the peptides were eluted into three fractions with 20 mM NH_4_HCO_2_, pH 10, containing 15, 30, and 90% ACN and dried in a rotary evaporator and stored at −80 °C until needed. 

### 2.5. Data Acquisition

SPS-MS3 TMT Data Acquisition on an Orbitrap Fusion Tribrid Mass Spectrometer:

RP-LC-MS/MS/MS was performed using a nanoACQUITY UPLC system (Waters Corporation, Milford, MA, USA) connected to an Orbitrap Fusion Tribrid (ThermoFisher Scientific, San Jose, CA, USA) mass spectrometer in the MS & Proteomics Resource of the Keck Foundation Biotechnology Resource Laboratory at Yale University. After injection, the samples were loaded into a trapping column (nanoACQUITY UPLC Symmetry C18 TRAP column, 180 µm × 20 mm) at a flowrate of 5 µL/min and then separated with a C18 column (nanoACQUITY column Peptide BEH C18, 75 µm × 250 mm). The compositions of mobile phases A and B were 0.1% formic acid in water and 0.1% formic acid in acetonitrile, respectively. Peptides were eluted with a gradient extending from 6% to 20% mobile phase B over 120 min and then to 40% mobile phase B for another 50 min at a flow rate of 300 nL/min and a column temperature of 37 °C. The data were acquired with the mass spectrometer operating in a top speed data-dependent mode with multinotch synchronous precursor selection (SPS)-MS3 scanning for TMT tags [27]. The full scan was performed over the range extending from 380–1580 m/z at an Orbitrap resolution of 120,000 at 200 m/z and automatic gain control (AGC) target value of 2 × 10^5^, with the maximum injection time set at 60 msec. Dynamic exclusion was enabled for a duration of 30 s. The most intense ions were selected above an intensity threshold of 5000 for collision-induced dissociation (CID)-MS fragmentation in the linear ion trap with collision energy of 35%. The isolation mode was set to quadrupole, and the isolation width was set to 1.6 m/z. The top 10 fragment ions for each peptide MS2 were notched out with an isolation width of 2 m/z and co-fragmented with higher-energy collision dissociation (HCD) at a collision energy of 65% to produce MS3 scans that were analyzed in the Orbitrap at a resolution of 60,000. The maximum injection time was set to 120 ms, and the AGC target value was set to 1 × 10^5^. 

### 2.6. Data Processing and Analysis 

Maxquant was used to process raw data files [28]. Default settings were used unless otherwise specified. Briefly, peptide spectrum match and protein false discovery rate (FDR) were set to a minimum of one unique peptide for identification and 1%, respectively. Fixed modifications were set to carbamidomethyl for cysteine, and variable modifications were set to methionine oxidation and N-terminal acetylation. Matches between runs were enabled with a default match time window of 0.7 min and alignment window of 20 min. The MS/MS spectra were matched against the mouse Uniprot FASTA database with canonical and isoform sequences. Search results and TMT reporter ion intensities were exported as text files. Statistical analysis was performed with Perseus 1.6.0.7 (Max Planck Institute of biochemistry, Martinsried, Germany) [29] and in-house R scripts (https://www.r-project.org/). Proteins identified by site, reverse, and potential contaminants were filtered in Perseus prior to further analysis. Proteins with missing values in any of the four D1 or four D2 biological replicates were filtered out before differential expression analysis.

## 3. Results

### 3.1. Purification of Nuclei

**Figure 1:**
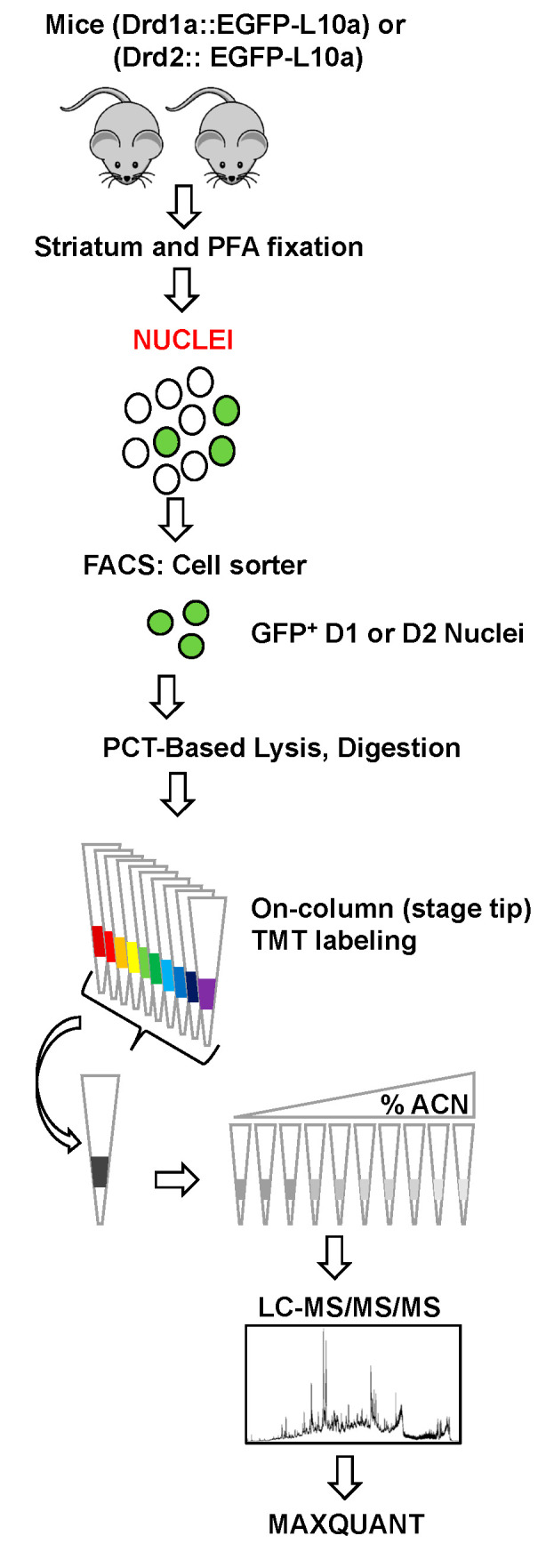
Proteomics workflow: Nuclei from striatal tissue from Drd1a:EGFP-L10a or Drd2:EGFP-L10a transgenic mice were sorted into microtubes. Lysis and trypsin digestion were then carried out using a PCT-MicroPestle (Pressure-cycling technology). After desalting and on-column TMT labeling in C18 StageTips, the samples were mixed and then subjected to pH 10 fractionation on sulfonated divinylbenzene (bSDB) packed StageTips. The resulting fractions were then analyzed by LC-MS/MS/MS with the raw data being processed by MaxQuant.

**Figure 2:**
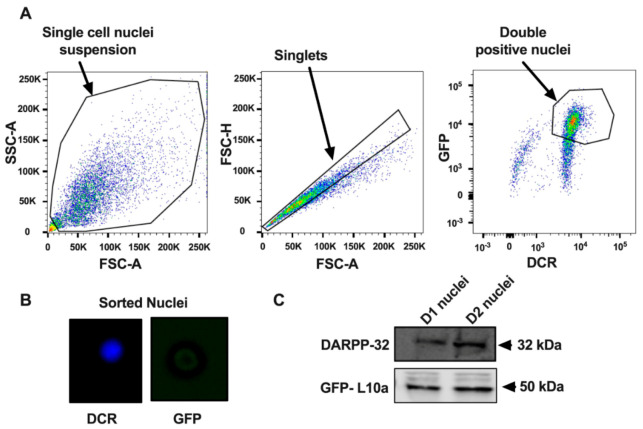
Fluorescence-activated sorting of nuclei from Drd1a:EGFP-L10a or Drd2:EGFP-L10a transgenic mice. (**A**) Flow cytometry gating strategies for sorting of D1 or D2 nuclei from wild-type (WT) mouse and Drd1a:EGFP-L10a or Drd2:EGFP-L10a transgenic mice. The double positive-isolated nuclei fraction is shown in the dot-plot of GFP fluorescence versus DyeCycle Ruby (DCR) fluorescence. DyeCycle Ruby binds to the DNA and aids the identification of singlets versus aggregated nuclei. The data from WT mice were used to set a threshold for the GFP signal background. FSC-A: Forward scatter area, SSC-A: Side scatter area, FSC-H: Forward scatter height. (**B**) After sorting, nuclei were verified by fluorescence microscopy and (**C**) Western blot (See Supplemental Materials for uncropped blots).

As depicted in the workflow in Figure 1 we used transgenic mice carrying bacterial artificial chromosomes (BACs) that express an EGFP-L10a fusion protein under the control of a cell-type-specific regulatory promoter; D1R (drd1a-EGFP mice) or D2R (drd2-EGFP mice). L10a is one of the proteins in the large subunit of the ribosome. Accumulation of EGFPL10a during ribosome assembly in the nucleolus imparts a bright green fluorescence that enables fluorescence-activated sorting of the corresponding nuclei (FANS) from the D1R or D2R-containing striatal MSNs. A similar isolation approach was used previously to study differential effects of cocaine on histone post-translational modifications in D1R versus D2R-containing MSNs [18]. In contrast to this earlier study [18] where isolated nuclei were briefly fixed with PFA, but more similar to another previous study [7], minced striata were exposed to a short fixation with 4% PFA (see Materials and Methods). After homogenization and sub-cellular fractionation, the resulting nuclei were subjected to FANS (Figure 2A) with DyeCycle Ruby (DCR) being used to label the nuclei. DCR is a DNA-intercalating dye that quantitatively binds to DNA [18,30]. Dot plots were then used to identify different types of nuclei based on their EGFP and DCR fluorescence intensity. Most nuclei displayed DNA fluorescence that was characteristic of single nuclei (singlets). The EGFP-fluorescent singlets were considered for further analysis while debris and aggregates were excluded (Figure 2A). A total of ~40,000 GFP-positive nuclei were isolated from each Drd1a:EGFP-L10a or Drd2:EGFP-L10a mouse brain with 2 mice (i.e., about 80,000 nuclei) being used for each of the four D1 and four D2 biological replicates. Finally, fluorescence microscopy was used to verify the integrity, purity, and identity of the FANS-purified nuclei. As shown in Figure 2B, 358-nm and 488-nm excitation lasers were used for detection of the DAPI (blue) signal for DNA and GFP signals (green), which is detectable after enlarging the figure, respectively. Anti-GFP or anti-DARPP-32 antibodies were used to detect GFP and DARPP-32, the latter being a striatal MSN-enriched protein present in both the nucleus and cytosol. Both proteins were present in sorted nuclei from either D1 or D2 MSNs (Figure 2C). 

### 3.2. Quantitative Proteomic Profiling of D1 and D2 Nuclei

**Figure 3:**
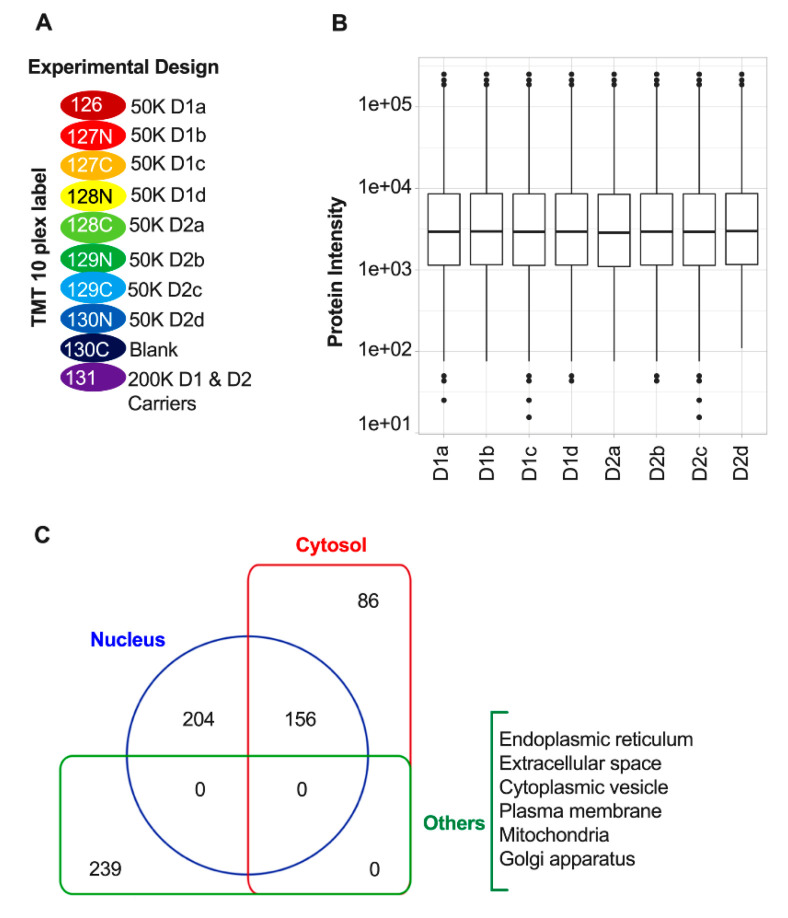
Experimental design and normalization. (**A**) Schematic for the design of the Tandem Mass Tag (TMT) 10-plex analyses of extracts from D1 and D2 nuclei. D1 or D2 nuclei in each biological replicate were FANS-sorted and 50,000 of the approximately 80,000 nuclei isolated from each of the two mice that constituted each of the four D1 and four D2 biological replicates (see Materials and Methods) were labeled with TMT reagents with the indicated reporter ions (RI). TMT label 130C and 131 represent blank and carrier channels, respectively (**B**) Protein abundance distribution of the MS3 data after quantile normalization. (**C**) Pie charts showing the cellular distribution of 685 proteins identified in quantitative proteomic analysis of D1 and D2 nuclei.

We adopted and further optimized a recent low input sample preparation protocol for MS/proteomic profiling of FACS-purified cells [25]. Our goals were to minimize sample handling, and to improve the lysis of fixed cells and the TMT labeling and fractionation of relatively low amounts of TMT-labeled tryptic peptides (~2 µg). The FANS-sorted D1 and D2 nuclei (i.e., 50,000 from the approximately 80,000 nuclei that were isolated from each pair of mice that constituted a biological replicate) were isolated in individual microTubes (Figure 3A), lysed, and the proteins were digested to peptides using Pressure-Cycling Technology (PCT) with MicroPestles and Caps in a Barocycler 2320 EXT. PCT with a MicroPestle, which is a disposable mechanical tissue homogenizer that fits inside the microTube sample container, was used to perform tissue homogenization and protein extraction by alternating cycles of ultra-high and low hydrostatic pressures [26]. Under repeated pulses of high hydrostatic pressure, the PCT-microTube walls collapse around the incompressible Teflon MicroPestle, creating a homogenizer-like environment. Additionally, the pressure cycle also acts directly on the sample, further disrupting membranes and solubilizing the proteins. These two concurrent actions contribute to more efficient tissue lysis and protein extraction, even for fixed tissues. After trypsin digestion, which also was carried out using PCT, each digest was then transferred to a C18 Stage tip for sample desalting and on-column TMT labeling that avoids an extra desalting step. After confirming the labeling efficiency, the 10 individual samples, which included one blank and one pooled carrier sample (Figure 3A), were mixed and fractionated by high pH SDB-RPS to reduce the complexity of the full proteome digests and increase the numbers of identified peptides and proteins. Sample losses were minimized by carrying out the SDB-RPS peptide fractionation in stage tips [25]. Isobaric labeling of peptides enables multiplexed peptide/protein identification and MS2/MS3 level quantitation for up to 16 samples using TMT [31,32,33]. Initially, the mass spectrometer carries out MS1 scans during which labeled peptides with the same sequence (and thus mass) from the 10 different samples (Figure 3A) appear as single unresolved, additive mass/charge (*m*/*z*) precursor ions. After CID MS/MS fragmentation of the precursor peptide ion, the top 10 fragment ions for each peptide MS2 were “notched out” and co-fragmented with HCD to produce MS3 scans that were analyzed in the Orbitrap. Maxquant was used to process the raw data with the MS3 spectra being used to quantify relative peptide and protein intensity.

Protein intensity was normalized first with quantile normalization and then the intensity of each protein was scaled by dividing by its mean intensity [31] (Figure 3B). On average, ~700 protein groups were identified in each sample with a 1% FDR for peptides and proteins. Across all samples 714 protein groups were quantified with 685 (95.9%) proteins with no missing values in any of the four biological replicates for either the D1 or D2 samples (Supplementary Table S1), and 29 (4.1%) proteins with from one (12 proteins, 1.7%) to six (6 proteins, 0.8%) missing values (Supplementary Table S2). Reflecting consistency in the biological replicates, the overall coefficient of variations (CVs) among the four biological replicates for the 685 proteins in the D1 and D2 samples were 14.6% and 16.5% respectively. Cellular component analyses using the GOCC database in Perseus was used to annotate the 685 proteins without any missing values. These analyses indicated that 360, 242, and 239 proteins were derived from either the nucleus, the cytosol, or other cellular compartments (i.e., endoplasmic reticulum, Golgi apparatus, cytoplasmic vesicle, plasma membrane, mitochondria, and extracellular space), respectively (Figure 3C, Supplementary Table S1). There were 156 proteins that are present in both the cytosol and nucleus.

### 3.3. Differentially Expressed Proteins in D1 and D2 Nuclei Fractions

**Figure 4:**
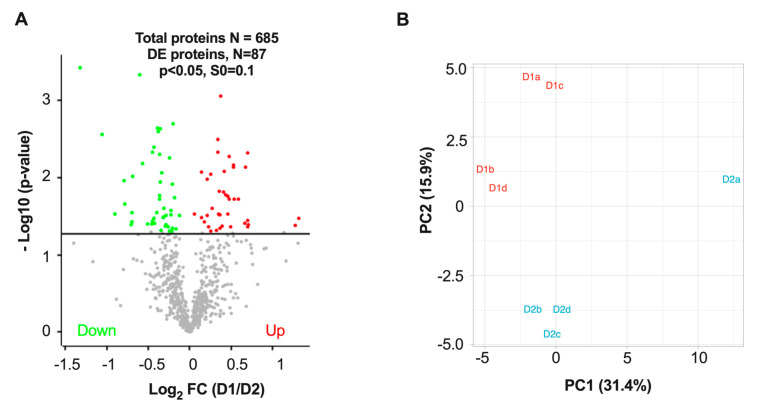
Differential protein expression between D1 versus D2 nuclei. (**A**) Volcano plot showing 87 differentially expressed (DE) proteins (i.e., those proteins above the solid line at 1.3 that corresponds to *p* < 0.05) out of the 685 quantified proteins). (**B**) Principal component analysis (PCA) of data after quantile normalization and mean scaling separates samples corresponding to D1 versus D2 nuclei. The percentage in parentheses is the proportion of the total variation for each principal component.

Since previous TRAP analyses revealed that dMSNs expressing high levels of D1R have different translational profiles than the iMSNs that express the D2R [21], we anticipated there also would be differences in the proteomes of nuclei from these two types of MSNs. Indeed, the TMT 10-plex analyses identified significant differences in relative protein expression between nuclei from these two different cell types. After log_2_ transformation and filtering to remove proteins that had missing values in any of the eight D1 and D2 samples, we quantified the relative D1/D2 expression ratios for 685 proteins (Supplementary Table S1). After quantile normalization, the relative protein intensity distributions appeared to be similar across all the samples (Figure 3B). After carrying out t-tests across all 685 protein groups, 87 (12.7%) were found to be significantly differentially expressed at *p* < 0.05 in D1 versus D2 nuclei fractions. As depicted in the volcano plot in Figure 4A and as summarized in Table 1, these analyses showed that 40 proteins were up-regulated and 47 proteins were down-regulated in D1 as compared to D2 nuclei. To better visualize differential D1 versus D2 protein expression, principal component analysis (PCA) was carried out using all 685 quantified proteins (Figure 4B). PCA analysis separated the data for the two cell type preparations into two mostly discrete clusters with the first and second components accounting for 31.4% and 15.9% of the overall variance, respectively.

### 3.4. Cluster Analysis of Differentially Expressed Proteins and Pathway Enrichment

**Figure 5:**
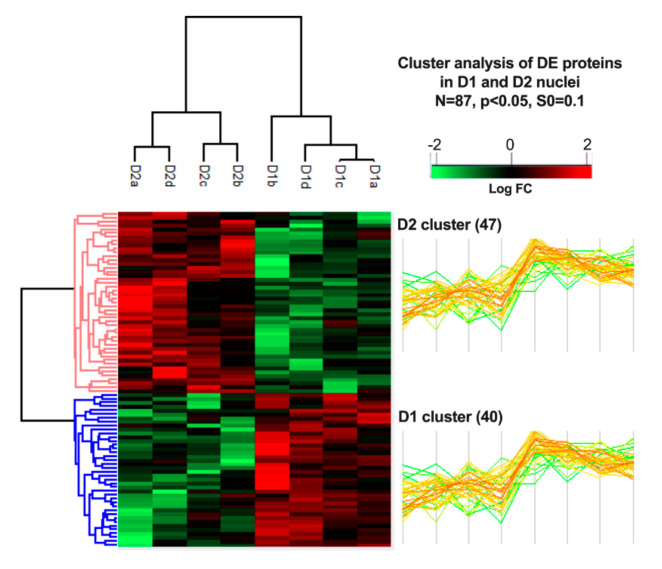
Heatmap of differentially expressed proteins in D1 and D2 cell types. Normalized intensities were Log_2_ transformed and were Z-scored prior to Euclidean distance-based hierarchical clustering with Perseus. Protein clusters D1 and D2 include 40 and 47 proteins, respectively.

Differential D1 versus D2 protein expression was highlighted further by carrying out hierarchical clustering of the 87 significantly differentially expressed proteins. These analyses were carried out using Perseus software in which sample groups use a Euclidean distance metric with average linkage. Preprocessing used k-means for data reduction purposes with Z-scores being normalized across sample groups prior to clustering. As shown in the heatmap in Figure 5, these analyses succeeded in separating all eight samples into D1 or D2 clusters containing the 40 proteins that are up-regulated and the 47 proteins that are down-regulated in D1 versus D2 nuclei preparations. We note that when all 685 proteins were included in the analyses that hierarchical clustering was unable to separate the eight samples into D1 and D2 clusters. 

**Figure 6:**
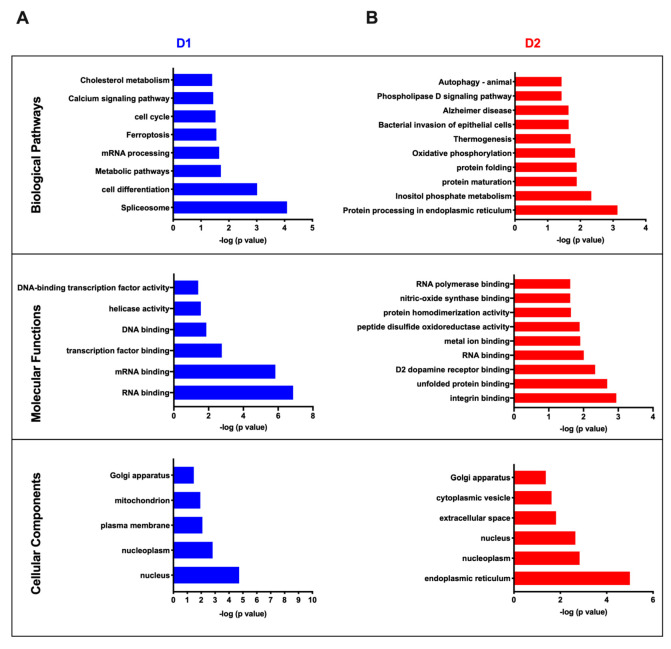
Go-annotation enrichment for cell type-specific clusters. (**A**) Enriched pathways and their *p*-values were obtained from the Fisher exact test < 0.05 in Perseus. D1 cell type displays protein expression changes broadly associated with spliceosome, cell differentiation, metabolic pathways, and other biological pathways listed in this figure. (**B**) D2 cell type displays protein expression changes broadly associated with protein processing, inositol phosphate metabolism, protein maturation, and other biological pathways listed in this figure.

To further characterize the 40 and 47 proteins that were up-regulated in nuclei preparations from either D1 or D2 MSNs respectively, these two groups of proteins were separately subjected to annotation enrichment analysis using Perseus with Fisher exact test *p* < 0.05 to identify positively enriched pathways, molecular functions, and cellular components (Figure 6A,B). The most significantly enriched cellular components in the D1 nuclei preparation were the nucleus and nucleoplasm. Consistent with this finding, the most significantly enriched molecular functions were those expected to be in the nucleus including RNA, mRNA, transcription factor, and DNA binding. Similarly, the two most significantly enriched pathways (i.e., cell differentiation and spliceosome) were both pathways that are located primarily in the nucleus. Although the nucleus and nucleoplasm were also among the three most significantly enriched cellular components in the D2 MSN preparation, the most significantly enriched cellular component in this nuclei preparation was the endoplasmic reticulum (Figure 6B). In agreement with the D1 nuclei preparation, RNA binding was also among the four most significantly enriched molecular functions in the D2 nuclei preparation (Figure 6B). The finding that D2 dopamine receptor binding was among the three most significantly enriched molecular functions in the D2 nuclei preparation provided validation of the source of the nuclei that were isolated from the D2 MSNs. Neither of the two molecular functions that were most significantly enriched (i.e., unfolded protein and integrin binding) in the D2 nuclei preparation were significantly enriched in the D1 nuclei preparation. Similarly, none of the four most significantly enriched D2 nuclei pathways (i.e., protein processing in endoplasmic reticulum, inositol phosphate metabolism, protein folding and maturation) were enriched in the D1 nuclei preparation.

### 3.5. Central Node Protein Networks in D1 and D2 Cell Types

**Figure 7:**
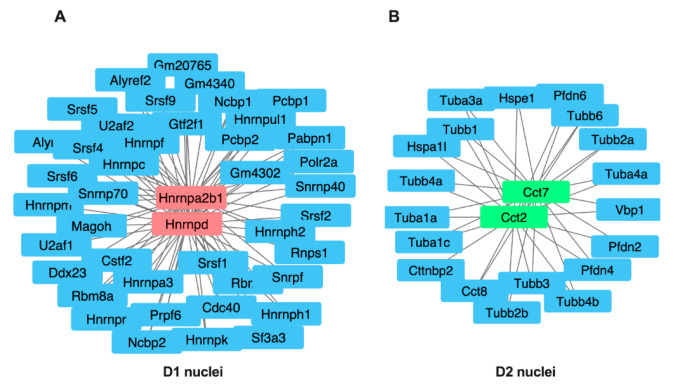
Central node protein networks. OmicsNet analysis of the differentially expressed proteins in D1 and D2 nuclei was used to generate protein–protein networks based on known experimental interactors. In the center are the central interactor proteins that are connected to genes encoding known interactors. These analyses identified Hnrnpd and Hnrnpa2b1 in D1 nuclei (**A**) and Cct2 and Cct7 in D2 nuclei (**B**) as central interactors.

Experimental protein–protein interaction networks were constructed for the two groups of 40 and 47 proteins that were enriched in D1 and D2 nuclei, respectively. Each of these groups of proteins was analyzed separately using OmicsNet, which identifies known interactors [34]. Interactions were based only on high-confidence STRING interactions for which there is experimental evidence. The identified networks were imported into Cytoscape and modified with the MCODE app to optimize their visual appearance [35,36]. As shown in Figure 7, the predicted protein–protein interaction networks for D1 and D2 nuclei generated two different central interactors for each cell type: Hnrnpd and Hnmpa2b1 in D1 and Cct2 and Cct7 in D2 nuclei preparations 

## 4. Discussion

To gain a better understanding of the functions and roles of D1 and D2 MSNs in a wide range of neurological disorders and diseases, we have optimized a FANS protocol for isolating nuclei from these individual cell types [18] and coupled it with a low input TMT 10-plex approach that uses on-column labeling for proteomic profiling of the relatively limiting amounts of protein that can be obtained from FANS-purified nuclei [25]. The genetically labeled nuclei from D1R- or D2R-expressing neurons were FANS-purified based on their having DyeCycle Ruby fluorescence expected for singlet nuclei and GFP fluorescence above a threshold background value that was based on nuclei isolated from wild-type mice. Western Blot analyses confirmed that both GFP and DARPP-32, the latter being found in both the nucleus and cytosol, were in the sorted nuclei from both D1 and D2 MSNs.

Since only limiting amounts of protein would be extracted from each of the aliquots of 50,000 FANS-sorted nuclei that were used for TMT labeling, the workflow that was devised incorporated approaches to improve: (1) extraction and digestion of proteins from the nuclei, (2) efficiency of TMT labeling and recovery of the labeled peptides, and (3) small-scale fractionation of the tryptic digests prior to LC-MS/MS/MS analyses. Since PCT has been shown to be a highly reproducible approach for extracting and digesting proteins from as few as 50,000 human cells and as little as 0.2–3 mg of mouse liver, heart and brain tissues [37], this technology was used for extracting proteins from the D1 and D2 MSN nuclei. PCT uses an instrument called a barocycler to apply ultra-high pressures up to 45,000 psi to a sample. Alternation between high and ambient pressures leads to physical disruption of complex tissue samples, extraction, and solubilization of proteins. PCT also enhances and accelerates proteolytic digestion of proteins into peptides [37]. PCT was carried out in a MicroPestle, which is a novel miniaturized, disposable mechanical tissue homogenizer that fits into the microtube sample container in the barocycler 2320 used in this study. The MicroPestle provides more efficient tissue lysis and protein extraction with smaller lysis and digestion volumes. Previous tests carried out with mouse liver, heart, brain, and human kidney tissues demonstrated that PCT-MicroPestle-extracted proteins yielded 20–40% more MS-ready peptide mass from all tissues tested with comparable high reproducibility as compared to the conventional PCT method [26]. 

On-column peptide labeling with TMT reduces the time for carrying out labeling from about 3 hrs to less than 10 min and the number of sample handling steps as compared to in-solution labeling because the desalting and labeling are carried out on the same resin [25]. On-column labeling of low microgram inputs of a murine B cell digest also has been shown to increase the efficiency and reproducibility of TMT labeling from 86+/−6% to 93+/−1% and it also provided a 27% increase in peptide spectral matches as compared to in-solution labeling [25]. Finally, small-scale fractionation of TMT-labeled peptides was incorporated into the protocol because it has been shown to improve depth of coverage for low input proteomics. For example, while single-shot MS/MS analyses of 1 µg of a murine B cell digest resulted in ~21 K peptide spectrum matches (PSMs), prior fractionation into three fractions increased the number of PSMs to about 39 K, 48 K, and 52 K for Stage Tips separations that were carried out with strong cation exchange (SCX), basic reversed phase C18, and basic sulfonated polystyrenedivinylbenzene (bSDB, pH 10) supports respectively [25]. The improved separation on both basic reversed phase modalities likely results from TMT labeling converting primary amines into amides, thus decreasing the positively charged character of tryptic peptides and their interactions with the SCX support [25]. 

We applied the low input proteomic-sample preparation workflow to nuclei preparations from either D1 or D2 MSNs freshly isolated from mouse striatal tissue. To our knowledge, this study provides the first proteome analyses of the two major MSN cell types from striatum. On average, ~700 protein groups were quantified with high reproducibility, as shown by the high replicate recall among the four D1 and four D2 biological replicates, i.e., 685 (96%) of the 714 proteins identified in all samples had no missing values in any of the four D1 or four D2 biological replicate samples. Cellular component analyses annotated all 685 protein groups that were identified across all eight TMT analyses without any missing values. These analyses indicated that 360 (53%) of the annotated proteins were found in the nucleus. The finding that the purified nuclei contained 325 proteins that are usually found only in the cytosol (86 proteins) and in other cellular compartments (239 proteins, e.g., endoplasmic reticulum, plasma membrane, Golgi apparatus, mitochondria, etc.) suggests that the gradient centrifugation and subsequent FANS separation of briefly fixed tissue may not have been sufficient to remove all non-nuclear proteins. 

Based on analyses of the 685 proteins that were quantified in all eight samples, 40 proteins were identified that were significantly (*p* < 0.05) up-regulated and 47 proteins were down-regulated in nuclei from D1 versus D2 MSNs. PCA carried out on all 685 quantified proteins separated the four D1 and four D2 samples into two mostly discrete clusters. Although we caution that with multiple test correction there were no significant differences in expression between proteins from D1 versus D2 nuclei, the observed differential expression of 87 proteins was highlighted further by hierarchical clustering analyses of these proteins. These analyses separated all eight samples into D1 and D2 clusters containing the 40 proteins that are up-regulated and the 47 proteins that are down-regulated in D1 versus D2 nuclei. To further characterize these two groups of differentially expressed proteins, each group of proteins was separately subjected to annotation enrichment analyses. Although the most significantly enriched cellular components (*p* < 0.01) in the D1 nuclear fraction were the nucleus, nucleoplasm, and plasma membrane; the most significantly enriched components in the D2 fraction were the endoplasmic reticulum, nucleoplasm, and nucleus. The appearance of endoplasmic reticulum proteins in the D2 nuclear fraction may result from the membranes of the endoplasmic reticulum being continuous with that of the outer nuclear membrane. The finding that D2 dopamine receptor binding is one of the molecular functions that was significantly (*p* < 0.01) enriched in the D2 nuclear fraction provides confirmation of the identity of the cell type of this nuclear fraction. Pathway enrichment analyses that were based on these 87 differentially expressed proteins suggested their association with addiction and neurological disorders. Hence, the phospholipase D signaling pathway, which has been linked to cocaine addiction and Alzheimer disease, was up-regulated in D2 cell type samples; while the calcium signaling pathway, which has been associated with both addiction and neurological disorders, was up-regulated in D1 cell type [38,39,40,41]. 

Construction of experimental protein–protein interaction networks for the two groups of 40 and 47 proteins that were enriched in D1 and D2 nuclei respectively identified two different central interactors for each cell type: Hnrnpd and Hnrnpa2b1 in D1 and Cct2 and Cct7 in the D2 nuclear fractions. Hnrnpd and Hnrnpa2b1 encode heterogeneous nuclear ribonucleoproteins (Hn D0 (UniProtKB: HNRPD_MOUSE) and A2/B1 (UniProtKB: ROA2_MOUSE) respectively that are located primarily in the nucleus [42]. Heterogeneous nuclear ribonucleoprotein (hnRNP) D0 binds RNA molecules that contain AU-rich elements (AREs) that are within the 3’-UTR of many proto-oncogenes and cytokine mRNAs. hnRNP D0 also binds to double- and single-stranded DNA sequences in a specific manner and functions as a transcription factor. Together with at least 20 other proteins hnRNP A2/B1 binds nascent pre-mRNAs and packages them into hnRNP particles that are involved in transcription, pre-mRNA processing, RNA nuclear export, sub-cellular location, mRNA translation, and stability of mature mRNAs. In oligodendrocytes and neurons hnRNP A2/B1 is involved in the transport of specific mRNAs to the cytoplasm. hnRNP A2/B1 also binds and protects single-stranded telomeric DNA sequences against endonuclease digestion. The two central interactors Cct2 and Cct7 in the D2 nuclear fractions encode the T-complex protein 1 subunits beta (UniProtKB: TCPB_MOUSE) and eta respectively that are components of the chaperonin-containing T-complex (TRiC) that assists the folding of ~10% of eukaryotic newly translated proteins in the cytoplasm [43]. Interestingly, TRiC is involved in axonal transport and it plays a neuroprotective role in neurodegenerative diseases including Huntington’s disease [44]. 

## 5. Conclusions

In summary, we report differential proteome analyses of nuclear preparations from D1 versus D2 MSNs and a novel workflow for low protein input TMT analyses of FANS-isolated nuclei from cell-type-specific populations of striatal MSNs. This method can now be used to improve our understanding of many neurological diseases including characterizing the short and long-term impact of drugs of abuse on the proteomes of these two neuronal populations. The importance of this novel workflow that now permits proteome analyses of D1 versus D2 MSNs was well demonstrated by comparing unpublished BacTRAP RNASeq data with the D1 and D2 MSN nuclear proteome analyses (both obtained from the same transgenic BacTRAP mouse lines). Based on a plot of the log2-fold-change between D1 and D2 MSNs for the 646 gene products that were common to both data sets, there was negative correlation (r = −0.10) between mRNA and protein expression levels (data not shown), thus highlighting the need to carry out proteomic measurements. Lastly, we note that one limitation of the current study is that its focus on the nuclear proteome requires that other components of the cellular proteome be discarded. To obviate this concern, we and others are currently exploring alternative approaches (e.g., LCM, controlled tissue fixation, organelle and sub-cellular fraction enrichment) using alternative fluorescent tags under cell-type-specific control.

## Figures and Tables

**Table 1:** List of significant differentially regulated proteins between D1 versus D2 nuclei (*p* < 0.05).

Up-Regulated (40)			Down-Regulated (47)		
Gene Name	Fold Change	*p*-Value	Gene Name	Fold Change	*p*-Value
***Tox4***	2.49116	0.0337605	***Coro2a***	0.400584	0.00037369
***Kctd20***	2.42119	0.0411815	***Hba***	0.481936	0.00274324
***Hnrnpab***	1.63754	0.0409344	***Efhd1***	0.535936	0.02929
***Snrpa1***	1.62469	0.0356868	***Septin 11***	0.578456	0.0109418
***Me3***	1.62296	0.00481903	***Dnajb11***	0.583221	0.0218579
***Eef1e1***	1.62035	0.0429891	***Sfxn5***	0.611712	0.0409574
***Elavl1***	1.59824	0.00725727	***Pam***	0.615849	0.037189
***Nup133***	1.58732	0.0387323	***Dlg4***	0.616571	0.0285791
***Vdac3***	1.50586	0.0188459	***Sacm1l***	0.619851	0.00970264
***Lsm6***	1.45014	0.0190832	***Aldoc***	0.658441	0.00046328
***Zfp292***	1.43915	0.00725454	***Ncam1***	0.676135	0.00650359
***Hnrnpa1***	1.43844	0.00683687	***Sfxn3***	0.703261	0.0397627
***D1Pas1***	1.40947	0.0434996	***Rps11***	0.726778	0.0394326
***Eftud2***	1.39301	0.00532517	***Cct2***	0.732122	0.0366409
***Hnrnpa0***	1.3922	0.0188692	***Pabpn1***	0.733746	0.0046371
***Celf2***	1.37592	0.0292762	***Pgap1***	0.740795	0.00399328
***Hnrnpdl***	1.37127	0.0172432	***Gabarapl2***	0.742762	0.0333492
***Syncrip***	1.35272	0.0166647	***Dctn3***	0.74543	0.0392584
***Rbmx***	1.33812	0.00827693	***Rpl24***	0.761663	0.0281739
***Purb***	1.32497	0.0154027	***Cct7***	0.763964	0.00226032
***Pura***	1.30983	0.0424454	***Slc4a4***	0.768653	0.00254452
***Hnrnpd***	1.29834	0.00088056	***Ppib***	0.777091	0.0189307
***Npm1***	1.29117	0.0299636	***Slc25a3***	0.777111	0.00503528
***Ptbp2***	1.29086	0.0454053	***Pdia6***	0.778812	0.0170188
***Tomm70***	1.28277	0.0149869	***Slc25a5***	0.780524	0.0112892
***Hnrnpl***	1.27938	0.0301104	***Vps35***	0.780705	0.00233104
***Ddx17***	1.2712	0.0293465	***Sec61a1***	0.788051	0.0480851
***Eif4a3***	1.26848	0.00472075	***Cct8***	0.789875	0.00857058
***Gnal***	1.26502	0.00318639	***Apmap***	0.800946	0.0324994
***Atp6v1b2***	1.24752	0.0477972	***Slc3a2***	0.805497	0.0251531
***Srrm2***	1.21029	0.0249769	***Tmpo***	0.807824	0.0318018
***Rbmxl1***	1.19467	0.00896132	***Asrgl1***	0.815023	0.0427078
***Hnrnpu***	1.19465	0.0490688	***Dctn2***	0.819532	0.0305211
***Erh***	1.17264	0.0428214	***Ganab***	0.820572	0.0307506
***Ruvbl1***	1.16093	0.0104841	***Plch1***	0.821746	0.0407058
***Hnrnpa2b1***	1.15681	0.0310403	***Ckap4***	0.835119	0.0435046
***Ndufa6***	1.13342	0.0371455	***Dnm2***	0.845664	0.0485707
***Prpf19***	1.10611	0.0325835	***Calr***	0.847391	0.0055328
***Cplx1***	1.10327	0.00840265	***Sv2a***	0.854525	0.0302238
***Vdac2***	1.04319	0.0298126	***Glud1***	0.857564	0.0264393
			***Hadha***	0.858226	0.0489431
			***P4hb***	0.864091	0.0451958
			***Thy1***	0.866795	0.012009
			***Rab6a***	0.870134	0.00202572
			***Ctsd***	0.882804	0.0180612
			***Dnm1***	0.889323	0.0456554
			***Hpcal4***	0.919234	0.0307775

## Supplementary Materials

The following are available online at https://www.mdpi.com/2227-7382/8/4/27/s1, Figure S1: Western blot analysis of D1 and D2 sorted nuclei with DARPP-32 antibody, Figure S2: Western blot analysis of D1 and D2 sorted nuclei with GFP antibody, Table S1: All quantified proteins with no missing values in D1 and D2 biological replicates, Table S2: List of proteins with missing values in D1 and D2 biological replicates.
