# Supplementary figures and images for: Differential Protein Expression in Striatal D1- and D2-Dopamine Receptor-Expressing Medium Spiny Neurons
Source: Proteomes. 2020 Oct 13;8(4):27. doi: 10.3390/proteomes8040027 (PMC7709116; doi:10.3390/proteomes8040027)

D1 D2

DARPP-32 (32kDa)

75kDa

25kDa

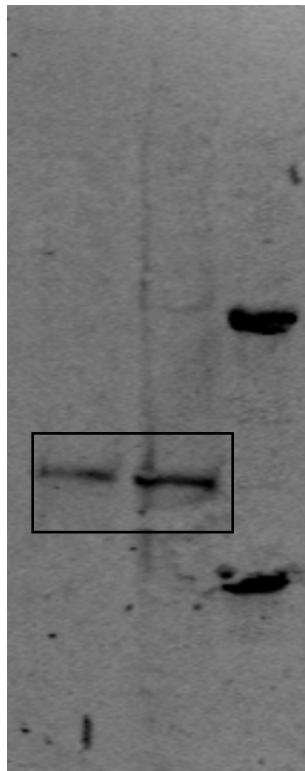

Supplement: Supplementary file 1 [file proteomes-08-00027-s001.zip › Figure 2_Western Blot_DARPP-32.pdf]

**D1 D2**

**250 kDa**

**150 kDa**

**75 kDa**

**50 kDa**

**37 kDa**

**25 kDa**

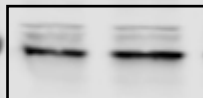

**GFP L10a (50 kDa)**

Supplement: Supplementary file 1 [file proteomes-08-00027-s001.zip › Figure 2_Western Blot_GFP-L10a.pdf]
